# An iteration model for identifying essential proteins by combining comprehensive PPI network with biological information

**DOI:** 10.1186/s12859-021-04300-7

**Published:** 2021-09-08

**Authors:** Shiyuan Li, Zhen Zhang, Xueyong Li, Yihong Tan, Lei Wang, Zhiping Chen

**Affiliations:** 1 College of Computer Engineering and Applied Mathematics, Changsha University, Changsha, 410022 China; 2 Hunan Province Key Laboratory of Industrial Internet Technology and Security, Changsha University, Changsha, 410022 China; 3 College of Electronic Information and Electrical Engineering, Changsha University, Changsha, 410022 China

**Keywords:** Essential proteins, Orthologous proteins, Multiplex biological networks, Subcellular localization information

## Abstract

**Background:**

Essential proteins have great impacts on cell survival and development, and played important roles in disease analysis and new drug design. However, since it is inefficient and costly to identify essential proteins by using biological experiments, then there is an urgent need for automated and accurate detection methods. In recent years, the recognition of essential proteins in protein interaction networks (PPI) has become a research hotspot, and many computational models for predicting essential proteins have been proposed successively.

**Results:**

In order to achieve higher prediction performance, in this paper, a new prediction model called TGSO is proposed. In TGSO, a protein aggregation degree network is constructed first by adopting the node density measurement method for complex networks. And simultaneously, a protein co-expression interactive network is constructed by combining the gene expression information with the network connectivity, and a protein co-localization interaction network is constructed based on the subcellular localization data. And then, through integrating these three kinds of newly constructed networks, a comprehensive protein–protein interaction network will be obtained. Finally, based on the homology information, scores can be calculated out iteratively for different proteins, which can be utilized to estimate the importance of proteins effectively. Moreover, in order to evaluate the identification performance of TGSO, we have compared TGSO with 13 different latest competitive methods based on three kinds of yeast databases. And experimental results show that TGSO can achieve identification accuracies of 94%, 82% and 72% out of the top 1%, 5% and 10% candidate proteins respectively, which are to some degree superior to these state-of-the-art competitive models.

**Conclusions:**

We constructed a comprehensive interactive network based on multi-source data to reduce the noise and errors in the initial PPI, and combined with iterative methods to improve the accuracy of necessary protein prediction, and means that TGSO may be conducive to the future development of essential protein recognition as well.

## Background

Numerous studies have shown that essential proteins play important roles in human biological processes. The lack of essential proteins will affect cell growth and development seriously, and the functions of the protein complexes will be lost as well. Essential protein prediction is not only of great significance to the researches on life science, but also able to provide valuable information to the treatment of diseases and the design of new drugs [1–4]. Traditionally, essential proteins are identified by medical experiments, such as RNA interference (RNAi) [5, 6] and gene knockout [7]. Chen et al. described a method for identifying essential genes of Streptococcus sanguis SK36 strain using whole-genome deletion mutations [8]. Ji et al. used antisense technology to construct a controllable gene expression system, and conducted a comprehensive genome analysis of Staphylococcus aureus, an important human pathogen [9]. In [10, 11], the necessity of each gene in the genome is analyzed by the method of sequencing the targeted insertion site of the transposon. However, these biological experiments are not only time-consuming, but also costly and inefficient. Hence, automated and accurate detection methods become necessary. Up to now, many computational models for identifying essential proteins have been developed successively. For instance, Yu et al. found the correlations between bottlenecks and essential proteins, where bottlenecks were defined as proteins with high degrees of centrality [12]. Based on the modular nature of a protein essentiality, Li Min et al. proposed a calculation method to identify essential proteins based on local average connection [13],and they also proposed a new model by adopting a new protein network recognition method based on topological potential [14], the basic idea is to treat each protein in the network as a material particle, generate a potential field around it, and calculate the topological potential of each protein to determine the importance of the protein. Jeong et al. introduced the central lethal rule to estimate the connection between network topology and essential proteins [15]. From then on, based on the concept of centrality, a lot of different methods, including the Degree Centrality (DC) [16], Information Centrality (IC) [17], Eigenvector Centrality (EC) [18], Subgraph Centrality (SC) [19], Betweenness Centrality (BC) [20], Closeness Centrality (CC) [21] and Neighbor Centrality (NC) [22], have been designed successively. However, although these centrality-based methods can improve the efficiency of traditional biological experiments effectively, their recognition abilities are still not very satisfactory, since there are lots of noises such as the false negatives and the false positives existing in the PPI networks [23, 24]. Therefore, in order to further improve the performance of identification models, biological information data including GO (Gene Ontology) statement annotations, gene expression profiles, subcellular data and protein domain data have been integrated with the PPI networks to identify essential proteins. For example, by calculate the co-expression and edge clustering coefficient between nodes, integrating PPI networks with gene expression data, Li et al. established a prediction method called Pec [25] to infer potential essential proteins. Zhang et al. proposed a computational model named CoEWC, integrates the clustering coefficient and gene co-expression properties of nodes, capture the common features of essential proteins in both date hubs and party hubs, and achieved good prediction performance [26]. Zhao et al. designed a model called POEM to predict essential proteins, POEM combines network topology with gene expression profiles to reduce the negative impact of PPI noise. Unlike other methods, POEM pays more attention to predicting the essential biological modules and uses calculation methods to determine the date hubs and party hubs [27]. Zhao et al. proposed that only constructing a single network will easily ignore the differences in biological characteristics and functional relevance, and conceal the inherent properties of heterogeneous data. Hence, Zhao et al. combined PPI with multiple biological data to construct a heterogeneous network to predict essential proteins [28].

The GO database is the largest source of information about gene function in the world [29], which has often been adopted to mine functional similarities between proteins. For instance, Kim et al found that it can improve the prediction performance of models by adopting the informational GO terms to prune the PPI networks [30]. Zhang et al. combined PPI with GO annotations and protein domain information to construct a three-dimensional tensor, and infer essential proteins through an extended HITS model [31], and got better performance. The meta-heuristic algorithm has the characteristics of high robustness, low complexity, and good optimization. Inspired by this, Lei et al. applied the intelligent evolutionary optimization algorithm to design the model and proposed a new method for predicting essential proteins in PPI networks based on artificial fish swarm optimization [32]. Zhang et al. defined a new measurement method for characterizing subcellular location information, and based on data fusion, proposed a new predictive model TEGS [33, 34]. Lei et al. designed a model called RSG through combining the RNA-seq data instead of the gene expression data with the GO annotation and subcellular localization to identify essential proteins [35], which is not only based on connectivity, but also considers co-expression level and functional similarity to measure protein importance.

Machine learning has also been applied in the field of essential protein identification. By using features from DNA and protein sequence data, Zhang et al. proposed a deep learning-based network embedding method to automatically learn features and use the features to train deep neural networks to predict human essential genes [36]. Zeng et al. proposed the Ess-NEXG model, which used RNA-seq, subcellular localization, orthology and other information to construct a reliable weighting network, and captured topological features through node2vec, and finally used a classifier to make predictions [37].

Considering that essential protein is more conservative than non-essential proteins in evolution, Peng et al. proposed an iterative method named ION to predict essential proteins by integrating orthology with PPI network [38]. Zhang et al. introduced a prediction method called OGN, in addition to the common topological attributes and co-expression probability of protein nodes in the date hubs and the party hubs, OGN adds orthologous scores to integrate the calculation of protein importance scores [39]. Lei et al. designed a method called PCSD for identifying essential proteins based on the degree of protein participation in protein complexes and the density of sub graphs [40]. Li et al. developed a prediction model called NCCO to identify potential essential proteins by extending the Pareto optimal consensus model (EPOC) [41]. Zhang et al. designed a dynamic PPI network (FDP). First, based on each time point, construct a series of active PPI networks, and then merge them one by one according to the similarity between the networks. Finally assign ranking scores to protein in consideration of homology and topological properties [42]. In our previous work, an iterative method called CVIM was proposed, which first integrates the topological characteristics of the PPI network based on the entropy weight method, and finally uses an iterative model to calculate and predict essential proteins based on orthologous information [43].

In this paper, different from above models, a novel centrality-based method called TGSO is proposed by combining biological essence data including the gene expression data, the orthologous information and the subcellular localization data with the topological information in a newly constructed comprehensive PPI network. In TGSO, a new centrality-based method named DBN (Density between nodes) is designed first to calculate the node density in complex networks, which can characterize the physical structure association between nodes in a complex network, and then, based on DBN, a protein aggregation degree interaction network (ADN) can be constructed. Next, by adopting the Pearson correlation coefficient to measure protein co-expressions based on the gene expression data, a protein co-expression interaction network (CEN) can be constructed. Moreover, based on the subcellular localization data, a protein co-localization interaction network (CLN) can be obtained as well. Hence, through integrating these three kinds of interaction networks, a comprehensive PPI network (PCIN) can be constructed. Finally, based on the newly obtained comprehensive PPI network, an iterative method called TGSO is designed to predict potential essential proteins by using the orthology information as the initial scores of proteins. In order to estimate the identification performance of TGSO, intensive experiments have been implemented, and experimental results show that TGSO can achieve more satisfactory prediction performance than state-of-the-art competitive prediction models such as DC [16], IC [17], EC [18], SC [19], BC [20], CC [21], NC [22], PEC [25], CoEWC [26], POEM [27], ION [38], TEGS [34] and CVIM [43] based on two kinds of different databases separately.

## Method

**Fig. 1:**
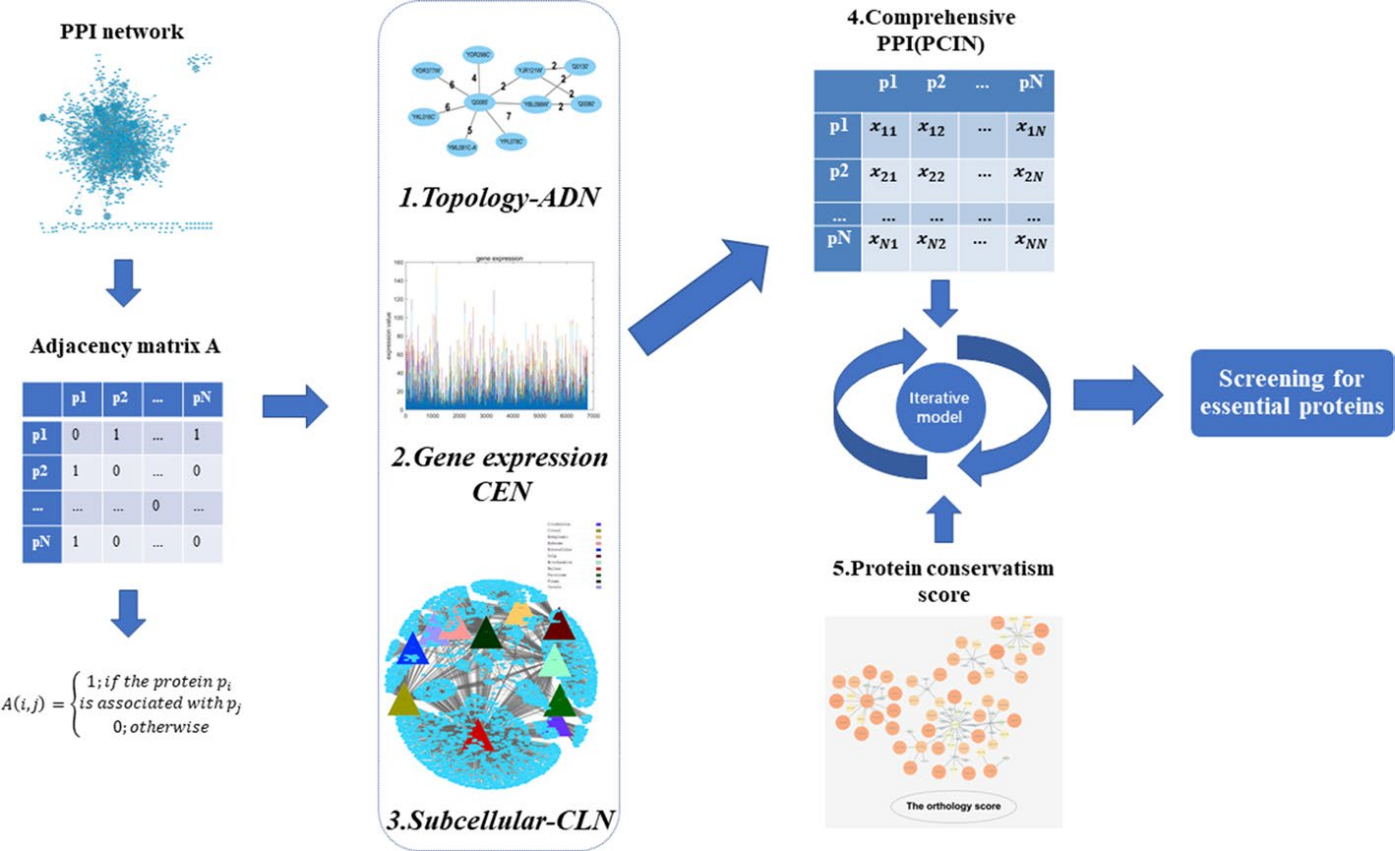
Flow chart of TGSO. (All the subgraphs in this figure were created by the first author using the open source software Cytoscape in combination with existing experimental data, without any borrowing.) The initial PPI, combined with subcellular localization and gene expression data as well as network topology information, was integrated to obtain the comprehensive protein interaction network, and the network and protein conservative score were put into the iterative model to obtain the final required protein score

As illustrated in Fig. 1, the procedure of TGSO mainly includes the following five steps:

**Step 1:** Construction of the ADN (the protein Aggregation Degree interaction Network).
**Step 2:** Construction of the CEN (the protein Co-Expression interaction Network).
**Step 3:** Construction of the CLN (the protein Co-Location interaction Network).
**Step 4:** Construction of the PCIN (the Protein Comprehensive Interaction Network).
**Step 5:** Construction of the TGSO.

The $G=(V,E)$ represents the PPI network downloaded from database *D.* Where $V=\{p_1, p_2,..., p_N\}$ is the set of protein nodes, and *E* is the set of edges in the network. As shown in Fig. 1, matrix $A=(a_{ij})_{N*N}$ represents the adjacency matrix of the protein, where there is $a_{ij}=1$, if and only if there exists an edge $e(p_i, p_j)$ between $p_i$ and $p_j$ in *E*, otherwise there is $a_{ij}=0$, the *N* represents the total protein amount.

### Construction of the ADN

Recent researches show that the degrees of connections between essential proteins are often higher than that between non-essential proteins [44], and essential proteins can form tightly connected molecular modules [33]. Hence, based on the modular nature of key proteins, for each edge *e*(*u*, *v*), we can design a local metric called DBN (Density between nodes) to measure the interaction between them in the original PPI network *G* as follows:

1 $$\begin{aligned} DBN(u,v) = \frac{|{NG(u)} \cap {NG(v)}+1|}{min(|NG(u)|,|NG(v)|)} \end{aligned}$$

Here, $NG(u)=\{v|\exists e(u,v) \in {E},v\in V\}$, represents the set of neighboring nodes of the protein node *u* in *G*, and |*NG*(*u*)| is the total number of neighboring nodes of the protein node *u* in *G*. According to above formula (1),we can obtain a new matrix DBN, on this basis can construct a new weighted PPI network, which is define as protein Aggregation Degree interactive Network (ADN).

### Construction of the CEN

Gene expression refers to the process of synthesizing genetic information from genes into functional gene products. Gene expression products are usually proteins, but the expression products of non-protein coding genes such as transfer RNA (tRNA) or small nuclear RNA (snRNA) genes are functional RNA. Over a period of time, there may be similar expressions between essential proteins. According to the studies of Horyu et al. [45], it was found that the Pearson correlation coefficient (PCC) is suitable for measuring the similarities between gene expression profiles. Hence, based on the concept of PCC, for any a pair of proteins *u* and *v*, we can calculate the similarity between them as follows:

2 $$\begin{aligned} PCC(u,v) =\frac{1}{n-1}\sum _{i=1}^n \biggr (\frac{{Exp(u,i)}-\overline{Exp(u)}}{\sigma (u)}\biggr )\biggr (\frac{{Exp(v,i)}-\overline{Exp(v)}}{\sigma (v)}\biggr ) \end{aligned}$$

Here, *Exp*(*u*, *i*) is the expression level of the protein *u* on the *i-th* time node, and for any given protein *u*, its expression information on a series of n different time nodes constitutes a vector $Exp(u)=\{Exp(u,1),Exp(u,2),...,Exp(u,n)\}$. In addition, $\overline{Exp(u)}$ is the average expression value of the protein *u*, $\sigma (u)$ is the standard variance for gene expression of the protein *u*.

Existing studies illustrate that the essentiality of proteins is related to the proteins or genes themselves and the molecular modules they belong to [46, 47], and the essential complex biological module consists of a large number of essential proteins that are highly connected and shared between biological functions [48]. Based on these findings, for any a pair of proteins *u* and *v*, we can measure the interaction between them in the original PPI network *G* as follows:

3 $$\begin{aligned} Connection(u,v) =PCC(u,v)+\sum _{\varepsilon \in ({NG(u)}\cap {NG(v)})}PCC(u,\varepsilon )*PCC(v,\varepsilon ) \end{aligned}$$

Based on above formula (3), we can construct another weighted PPI network, namely, protein co-expression interaction network (CEN).

### Construction of the CLN

Researches show that protein interactions in human bodies tend to coexist in the same cell compartment or adjacent cell compartments [49]. And it has been demonstrated that the introduction of subcellular localization information is of great help in screening essential proteins [28, 34, 35].

**Fig. 2:**
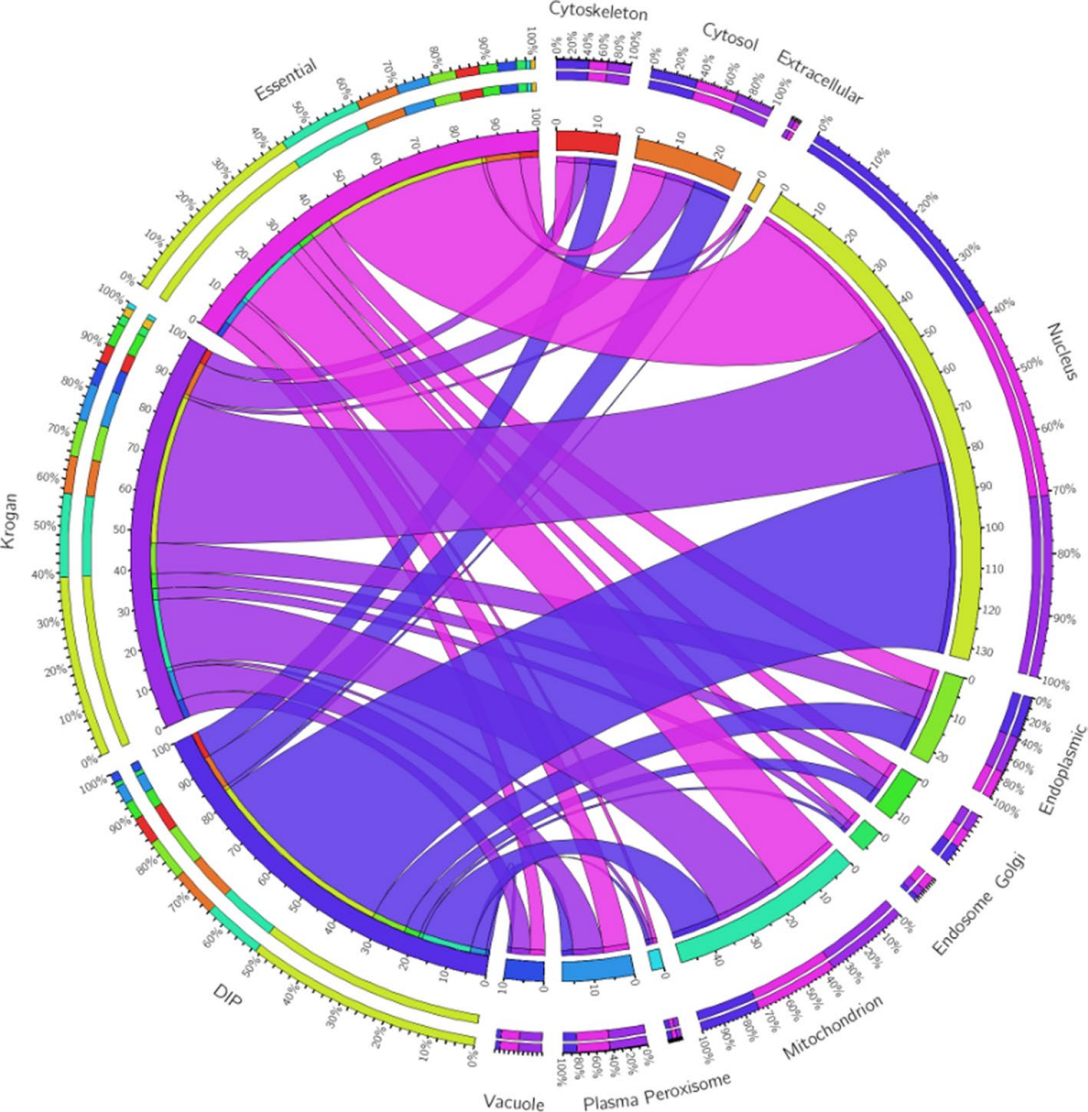
In the three data sets of Essential, Dip and Krogan, the percentage of Essential protein in 11 subcellular locations is represented by the thickness of the line, and the length of the outer ring represents the total proportion of Essential protein in this subcellular locations

As shown in Fig. 2, the cell nucleus has the largest number of essential proteins, while Extracellular and Peroxisome have only a small number of essential proteins. Moreover, individual subcellular sites had similar amounts of essential proteins in three different datasets. For example, Nucleus accounts for about 40% of essential proteins in DIP, Essential, and Krogan. Recent research discover that 76% of protein–protein interactions in yeast cells occur between identical subcells [50]. And in many cases, the product of complex functions is more important than the function of individual proteins, and essential proteins tend to form protein complexes to perform important functions together [46, 47]. Hence, in order to distinguish the importance of different subcellular localizations, for any given subcellular location *i*, we define the total number of subcellular species related to *i* as follows:

4 $$\begin{aligned} sub\_score(i) =\frac{sub(i)}{\sum _{k=1}^{N}{sub(k)}} \end{aligned}$$

Here, *sub*(*i*) represents the number of protein nodes associated with the subcellular location *i* in the database. Hence, for any give protein *u*, we can define its self-localization score as follows:

5 $$\begin{aligned} S\_score(u) = \sum _{i \in L(u)}sub\_score(i) \end{aligned}$$

Here, *L*(*u*) is a collection of all subcellular localizations possessed by *u*.

Based on above formula (5), for any a pair of proteins *u* and *v*, we can further obtain the co-localization score between them as:

6 $$\begin{aligned} colo\_sub(u,v) = \frac{|L(u)\cap L(v)|}{|L(u)\cup L(v)|}* \frac{S\_score(u)+S\_score(v)}{2} \end{aligned}$$

According to above formula (6),we can further construct a new weighted PPI network as the protein Co-Localization interaction Network (CLN).

### Construction of the PCIN

Based on above three kinds of newly constructed weighted PPI networks such as the AND, CEN and CLN, for any given protein *u*, we can obtain a unique score for *u* as follows:

7 $$\begin{aligned} LSG(u) = \sum _{v\in NG(u)}DBN(u,v)*(colo\_sub(u,v)+Connection(u,v)) \end{aligned}$$

According to above formula (7), for any two given proteins *i* and *j*, we can define a comprehensive interaction between them as follows:

8 $$\begin{aligned} PCIN(i,j) = {\left\{ \begin{array}{ll} LSG(i)/\sum _{k=1}^{N}LSG(k)&{} \quad if\quad i=j\\ min(LSG(i),LSG(j))/\sum _{k=1}^{N}LSG(k)&{} \quad Otherwise \end{array}\right. } \end{aligned}$$

where *N* is the total number of protein nodes.

### Construction of the TGSO

Peng et al. [38] found that the essentiality of protein is closely related to the degree of protein conservatism.

**Fig. 3:**
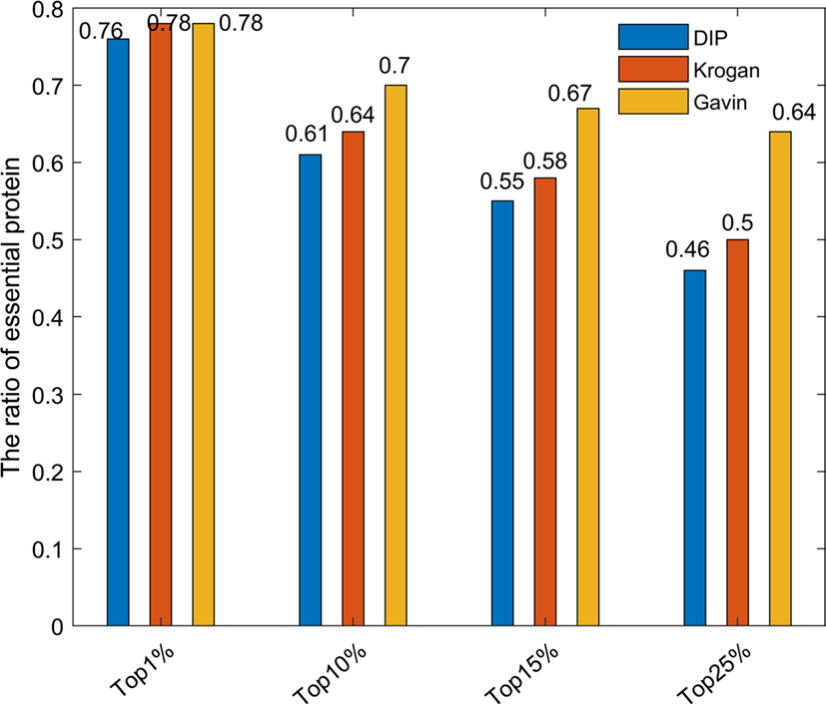
Conservative scores of performance in three yeast datasets

Figure 3 shows the brief results of using conservative scores alone to screen for essential proteins. The accuracy of this score reached 76% in the top 1% of the three databases. So the conservative score plays an important role in the recognition of essential protein, and we use this score as the initial score vector of protein.

For any given protein $u_i$, let *I*(*i*) denote its homology score, Eq. (9) can be obtained by referring to [38], where ${u_i \in V(i=1,2,...,N)}$

9 $$\begin{aligned} I(i) = \sum _{m \in S}T_i\quad where\quad T_i = {\left\{ \begin{array}{ll} 1&{} if\quad {u_i \in X_m}\\ 0&{} Otherwise \end{array}\right. } \end{aligned}$$

*S* is the set of reference organisms which is used to get orthologous information of node *V*. *s* denotes its element. |*S*| denotes the number of its elements. $X_s$ is a subset of node *V*. Its element has orthologs in organism *s*.

Then we can obtain the conservatism score $O\_score(i)$ corresponding to $u_i$ based on the original PPI network *G* as follows:

10 $$\begin{aligned} O\_score(i) = \frac{I(i)}{\sum _{k=1}^{N}{I(k)}} \end{aligned}$$

Based on above formula (10), for all *N* different proteins $p_1, p_2,..., p_N$ in *G*, then we can obtain their initial scores as follows:

11 $$\begin{aligned} P_0 = (O\_score(1), O\_score(2), ..., O\_score(i), ..., O\_score(N)) \end{aligned}$$

Finally, based on above newly obtained initial scores and the newly constructed weighted comprehensive PPI network PCIN, we use iteratively based on the weighted PageRank [51] to obtain the critical scores of all proteins in *G*:

12 $$\begin{aligned} P_{t+1} = (1-\alpha )*PCIN*P_t+\alpha *P_0 \end{aligned}$$

Here, the parameter $\alpha (0\leqslant \alpha \leqslant 1)$ is used to adjust the proportion of initial scores $P_0$ and last iteration scores $P_t$.

Based on the above descriptions, the general flowchart of our prediction algorithm TGSO can be mainly described as follows:

**Table:** 

**Algorithm: TGSO**
**Input**: Original PPI network $G = (V, E)$, subcellular location data, orthologous and gene expression data, the parameters $\gamma$ and *K*
**Output**: Top K percent of proteins sorted by the vector P in descending order
**Step1**: Constructing the ADN according to the formula (1);
**Step2**: Constructing the CEN according to the formula (3);
**Step3**: Constructing the CLN according to the formula (6);
**Step4**: Constructing the PCIN according to the formula (8);
**Step5**: Obtaining the initial score vector $P_0$ according to the formula (11);
**Step6**: Let $t=0$; Obtaining P1according to formula (12);
**Step7**: Let $t=t+1$; Obtaining $P_{t+1}$ according to formula (12);
**Step8**: Repeating Step7 until ${(||P_{t+1}-P_t||)}/|E|<\gamma$;
**Step9**: Sort proteins by the value of P in the descending order;
**Step10**: Output top K percent of sorted proteins.

## Result and analysis

### Experimental data

In order to estimate the identification performance of TGSO, in this section, we will compare it with 13 different state-of-the-art competitive prediction models illustrated in the following Table 1.

**Table 1:** A rough introduction to other algorithms

Algorithm	Network topology	Biological information
DC [16]	Degree Centrality	No
IC [17]	Information Centrality	No
EC [18]	Eigenvector Centrality	No
SC [19]	Subgraph Centrality	No
BC [20]	Betweenness Centrality	No
CC [21]	Closeness Centrality	No
NC [22]	Neighbor Centrality	No
Pec [25]	Edge clustering coefficient	Gene expression data
CoEWC [26]	Clustering coefficient	Gene expression data
POEM [22]	Degree Centrality, subgraph Edge clustering coefficient, closeness Centrality	Gene expression data
ION [38]	Edge clustering coefficient	Orthologous data
CVIM [43]	Average triangle, neighbor average triangle	Orthologous data, gene expression data
TEGS [34]	Edge clustering coefficient	Gene Ontology, subcellular localization Gene expression data

Since saccharomyces cerevisiae includes the most complete PPI data and rich biological information data, and is widely used to evaluate essential protein prediction models, we will first evaluate the performance of TGSO based on three saccharomyces cerevisiae related databases such as the DIP database [52], the Krogan database [53], and the Gavin database [54]. After filtering out repetitive interactions and self-interactions, as shown in the Table 2, we finally obtained a total of 5093 proteins and 24,743 interactions from the DIP database, 14,317 pairs of interactions between 3672 proteins from the Krogan database, and 1855 proteins and 7669 interactions from the Gavin database respectively.

**Table 2:** The detail information of the three PPI datasets

Dataset	Proteins	Interactions	Essential	Gene expression covers
DIP	5093	24743	1167	4981
Krogan	3672	14317	929	3610
Gavin	1855	7669	714	1827

Moreover, as a benchmark dataset for testing the accuracy of different identification models, a set of 1293 essential genes is derived from the MIPS [55], the Saccharomyces Genome Database(SGD) [56], the Saccharomyces Genome Deletion Project Database (SGDP) [57], and the Database of Essential Genes (DEG) [58] simultaneously. In addition, the gene expression data of Saccharomyces cerevisiae is obtained from the work proposed by Tu et al. [59], which contains 6777 gene products and 36 samples. The orthologous information is downloaded from the InParanoid database (Version 7) [60]. Besides, as illustrated in above Fig. 2, we derived eleven subcellular locations related to eukaryotic cells from the COMPARTMENTS database [61, 62] as well.

Finally, in order to evaluate the uniqueness and efficiency of TGSO, in this section, we will first adopt different measurements such as accuracy, jackknife, Precision Recall regression curve (PR-curves) and Receiver Operating Characteristic curve (ROC) to compare TGSO with 13 competitive prediction models shown in Table 1 comprehensively. And then, we will further estimate the effect of the parameter $\alpha$ on the performance of TGSO.

### Comparisons between TGSO and 13 representative methods

**Fig. 4:**
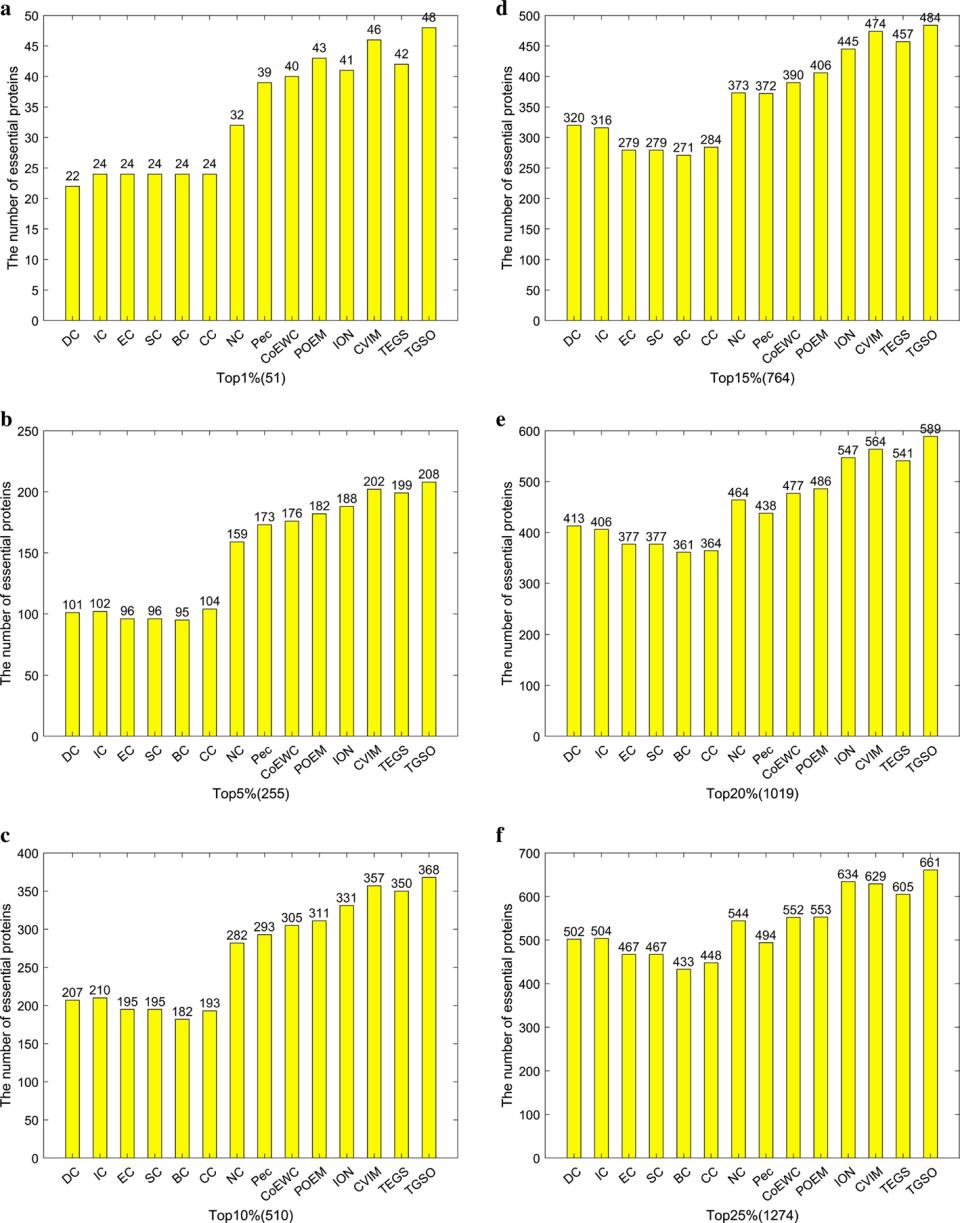
**a** Top 1% ranked proteins. **b** Top 5% ranked proteins. **c** Top 10% ranked proteins. **d** Top 15% ranked proteins. **e** Top 20% ranked proteins. **f** Top 25% ranked proteins. This figure illustrates the comparison of the number of essential proteins predicted by TGSO and 13 competing methods on the DIP dataset. The graph shows the number of truly essential proteins found by each method. The numbers in parentheses indicate the number of proteins ranked in each highest percentage

In this section, two kinds of datasets downloaded from the DIP database and the Krogan database separately are adopted to compare TGSO with 13 competitive prediction models illustrated in Table 1. And as a result, Fig. 4 and Table 3 show the comparison results based on the DIP database and the Krogan database respectively.

From observing Fig. 4, it is not difficult to see that in the top 1% (51) potential key proteins, TGSO has screened out 48 true essential proteins, with an accuracy rate of 94%. Among 5% (255) and 10% (510) candidate critical proteins, there are 208 and 368 true essential proteins having been identified by TGSO separately, with an accuracy rate of 82% and 72% as well.

Comparing with traditional centrality-based methods such as DC, IC, EC, SC, BC, CC and NC, the number of true essential proteins detected by TGSO has obvious advantages. Especially except NC, TGSO predicts twice as many truly essential proteins as other centrality methods in the top 1% and 5% of candidate essential proteins. And simultaneously, in the top 10% predicted essential proteins, while comparing with DC, IC, EC, SC, BC, CC and NC, the prediction accuracy of TGSO has increased by 77.78%, 75.24%, 88.72%, 88.72%, 102.2%, 90.67% and 30.5% respectively. Moreover, while comparing with methods that combined PPI networks with multiple biological data, such as Pec, CoEWC, ION, POEM and CVIM, TGSO can still achieve the highest prediction accuracy in any range from the top 1% to 25% of potential key proteins. Therefore, the results show that TGSO is the best predictor based on the DIP database.

From observing Table 3, it can be found that TGSO can achieve similar prediction performance based on the Krogan database. For instance, among the top 1% (37) candidate critical proteins, 35 true essential proteins have been detected by TGSO, with the accuracy rate of 95%, while in the top 15% (551) potential essential proteins, TGSO can still achieve the accuracy rate of 66.06%, which is 76.70% higher than that of the worst-performing CC, and 11.31% and 13.40% higher than that of the best-performing CVIM and TEGS respectively in these 13 tradition competitive models. Furthermore, with the increasing of candidate key proteins, the accuracy rate of all kinds of prediction models will decrease inevitably, but in the top 25%, the number of true essential proteins detected by TGSO has reached 515, which is still much higher than 479 detected by CVIM and 480 discovered by ION. Hence, we can draw the conclusion that TGSO can achieve the best identification performance based on both the Krogan database and the DIP database while comparing with these 13 competitive state-of-the-art prediction models.

**Table 3:** Number of essential proteins predicted by TGSO and 13 methods based on the Krogan database

Methods	Top1% (37)	Top5% (184)	Top10% (367)	Top15% (551)	Top20% (734)	Top25% (918)
SC	18	96	173	256	321	380
EC	20	91	173	251	317	378
BC	20	78	145	215	273	337
DC	20	78	145	215	273	337
IC	17	83	152	226	286	337
CC	13	68	142	206	262	326
NC	23	126	208	288	344	397
PEC	24	122	201	273	324	378
CoEWC	24	124	215	291	345	401
POEM	28	131	221	298	371	428
ION	31	133	238	317	392	480
CVIM	35	141	242	327	410	479
CVIM	32	142	246	321	392	449
TGSO	35	147	262	384	447	515

This table shows the commonalities and differences between TGSO and the 13 competitive methods in Table 1 based on the Krogan database

### Validation with jackknife methodology

**Fig. 5:**
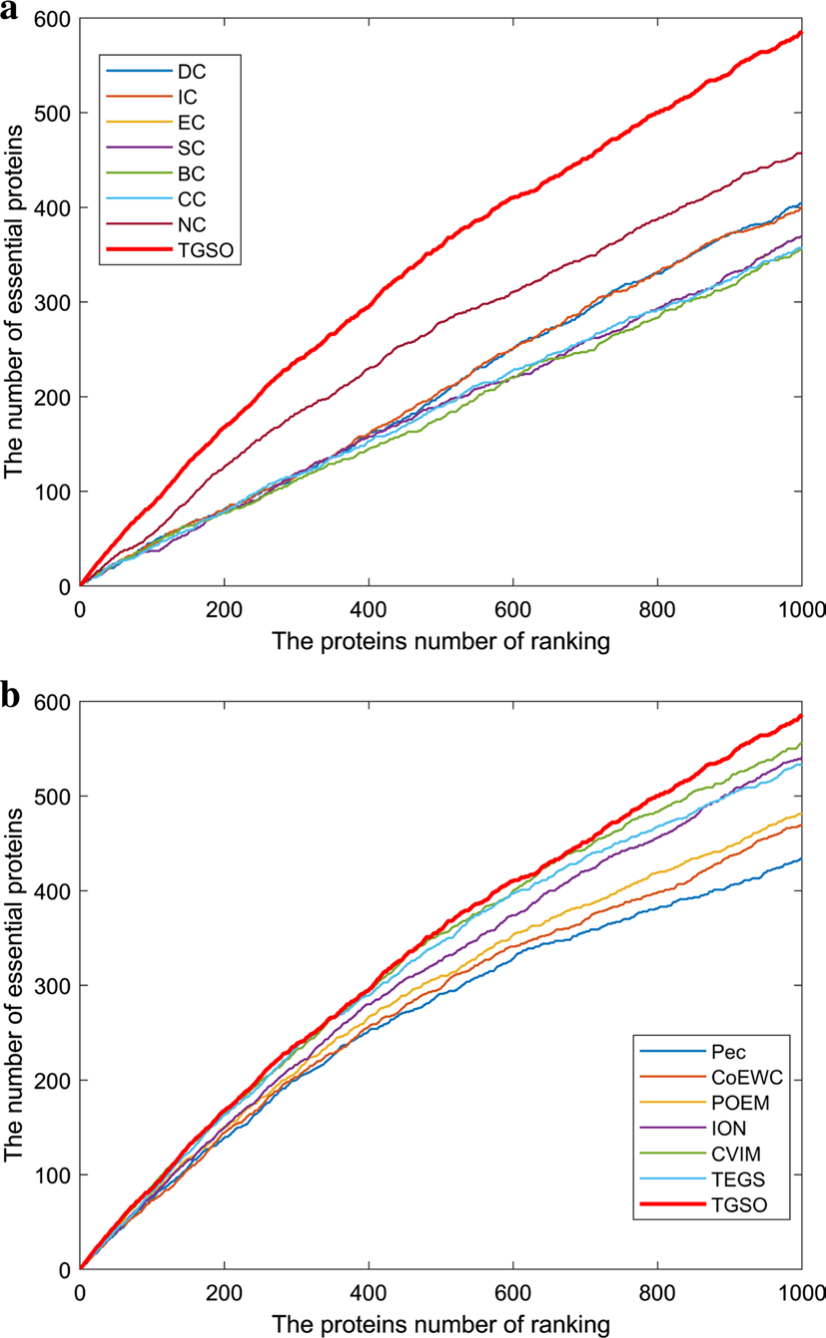
Comparison of Jackknife curves of TGSO and 13 other methods under the DIP database. **a** Comparison between TGSO and DC, IC, EC, SC, BC, CC, NC. **b** Comparison between TGSO and Pec, CoEWC, POEM, ION, CVIM, TEGS

**Fig. 6:**
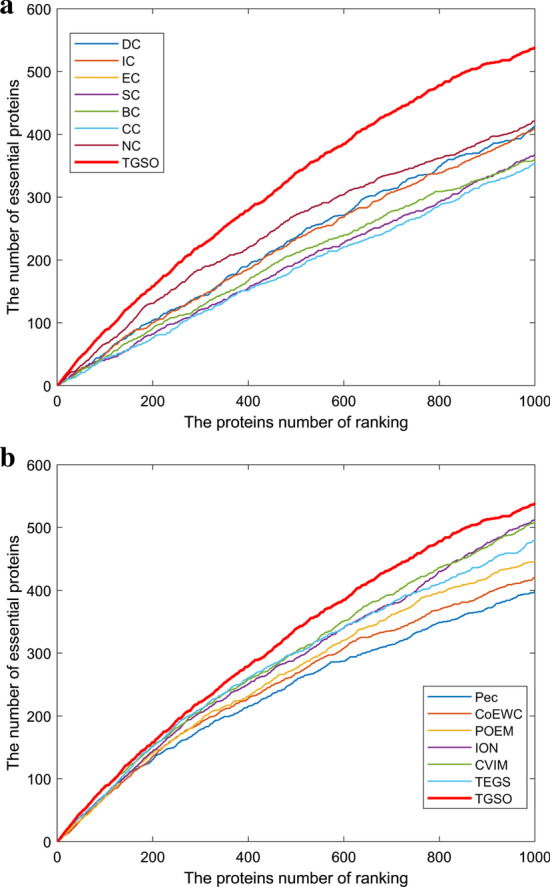
Comparison of Jackknife curves of TGSO and 13 other methods under the Krogan database. **a** Comparison between TGSO and DC, IC, EC, SC, BC, CC, NC. **b** Comparison between TGSO and Pec, CoEWC, POEM, ION, CVIM, TEGS

In order to evaluate the TGSO model more comprehensively and specifically, we extracted the top 1000 proteins sorted by importance score calculated by TGSO. TGSO’s ability to place experimentally validated essential proteins at the top of the ranked proteins was evaluated with Jackknife [63]. The X-axis represents the ordered proteome of an organism, arranged from left to right with the strongest prediction to the least prediction of importance. The Y axis is the cumulative count of essential proteins encountered as they traverse the ordered proteome from left to right. And as a result, Figs. 5 and 6 illustrate the comparison results. From observing Fig. 5a, TGSO can achieve better performance than these centrality-based methods including DC, IC, EC, SC, BC, CC and NC. Moreover, from observing Fig. 5b, the prediction performance of TGSO is significantly better than those multiple biological data based methods such as Pec, CoEWC, POEM and ION as well. Although there are some partial overlaps among TGSO and CVIM and TEGS, as the number of candidate key protein increases to about 600, the prediction performance of TGSO will become significantly higher than both CVIM and TEGS, which indicates that TGSO is superior to both CVIM and TEGS. In addition, from Fig. 6a, b, it is to see that TGSO can achieve better performance than all these 13 competitive methods. Especially, comparing with those methods that combined PPI networks with multiple biological data, while the number of candidate essential proteins reaches 300, TGSO can achieve much better performance than all these competitive methods simultaneously.

### Validation by precision–recall curves and ROC curves

**Fig. 7:**
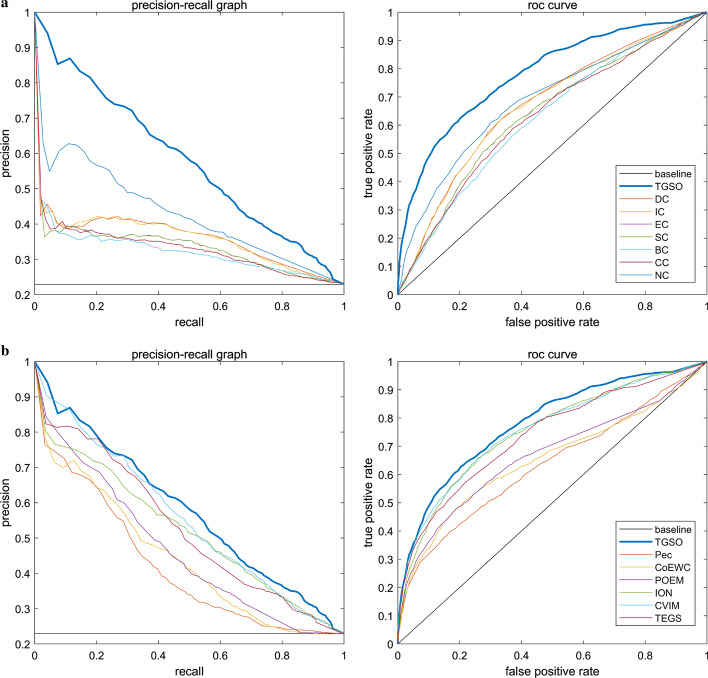
ROC curve and PR curve of various methods of PPI network based on the DIP database. **a** Comparison of TGSO with DC, EC, IC, SC, BC, CC and NC. **b** Comparison of TGSO with Pec, CoEWC, POEM, ION, CVIM and TEGS

**Fig. 8:**
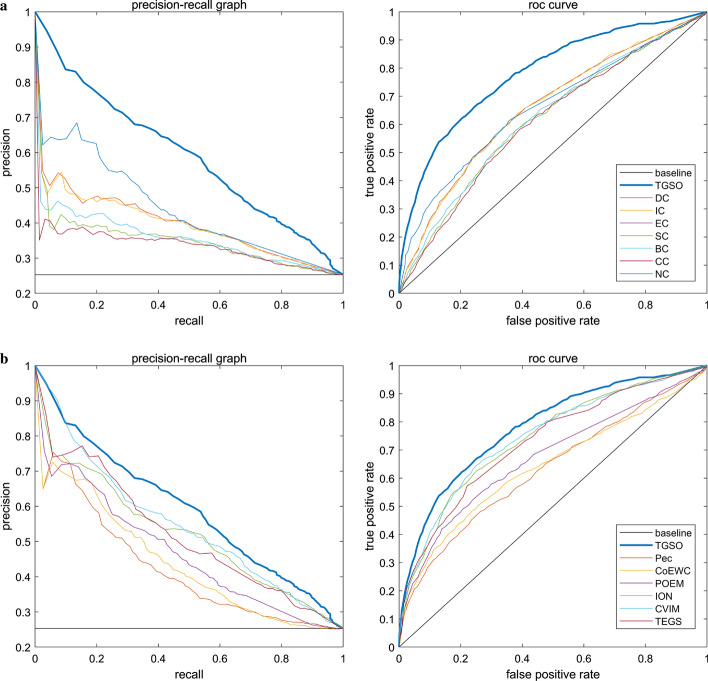
ROC curve and PR curve of various methods of PPI network based on the Krogan database. **a** Comparison of TGSO with DC, EC, IC, SC, BC, CC and NC. **b** Comparison of TGSO with Pec, CoEWC, POEM, ION, CVIM and TEGS

In this section, we will further use the receiver operating characteristic curve (ROC curve) to evaluate the performance of TGSO. Studies show that the larger the area under the ROC curve (AUC), the better the performance of the model, and if AUC=0.5, it means a random performance [64–66]. In the three kinds of yeast cell databases including the DIP, Krogan and GAVIN databases, the proportion of key proteins is very small, and the proportion of non-essential proteins and essential proteins is about 3 to 1. Studies show that while dealing with highly skewed datasets, the precision recall (PR) curve can provide more information about the performance of an algorithm [67]. Therefore, in this section, we will further adopt the PR curves to compare TGSO with 13 competitive methods. As shown in Figs. 7 and 8, the AUCs achieved by TGSO is much higher than that of competitive methods based on both the DIP database and the Krogan database. However, from observing Figs. 7b and 8b, we can find that the curves of TGSO and CVIM have a little overlap. Hence, in order to further evaluate TGSO and CVIM, we adopt the F1-score as well, and the comparison results are shown in Table 4.

From observing Table 4, not only the AUC achieved by TGSO is higher than those 13 competitive methods based on both the DIP database and the Krogan database, but also the F1-score achieved by TGSO is superior to those 13 competitive methods simultaneously. Therefore, it is reasonable to believe that TGSO has better performance than all these traditional state-of-the-art methods.

**Table 4:** The AUCs and F1-scores achieved by all methods based on the DIP and Krogan databases respectively

Method	AUC (DIP)	F1-score (DIP)	AUC (Krogan)	F1-score (Krogan)
TGSO	0.7813	0.5466	0.7808	0.5600
CVIM	0.7559	0.5217	0.7458	0.5411
ION	0.7522	0.5226	0.7413	0.5305
TEGS	0.7386	0.4959	0.7287	0.5148
POEM	0.6662	0.4528	0.6726	0.4704
CoEWC	0.6513	0.4528	0.6404	0.4476
Pec	0.6329	0.4062	0.6316	0.4264
NC	0.6879	0.4656	0.6584	0.4597
CC	0.6291	0.4143	0.6114	0.4282
BC	0.6250	0.4078	0.6248	0.4347
SC	0.6385	0.4233	0.6167	0.4309
IC	0.6657	0.4526	0.6573	0.4603
EC	0.6384	0.4235	0.6169	0.4308
DC	0.6705	0.4524	0.6583	0.4588

### Difference analysis of TGSO and 13 competitive methods

**Fig. 9:**
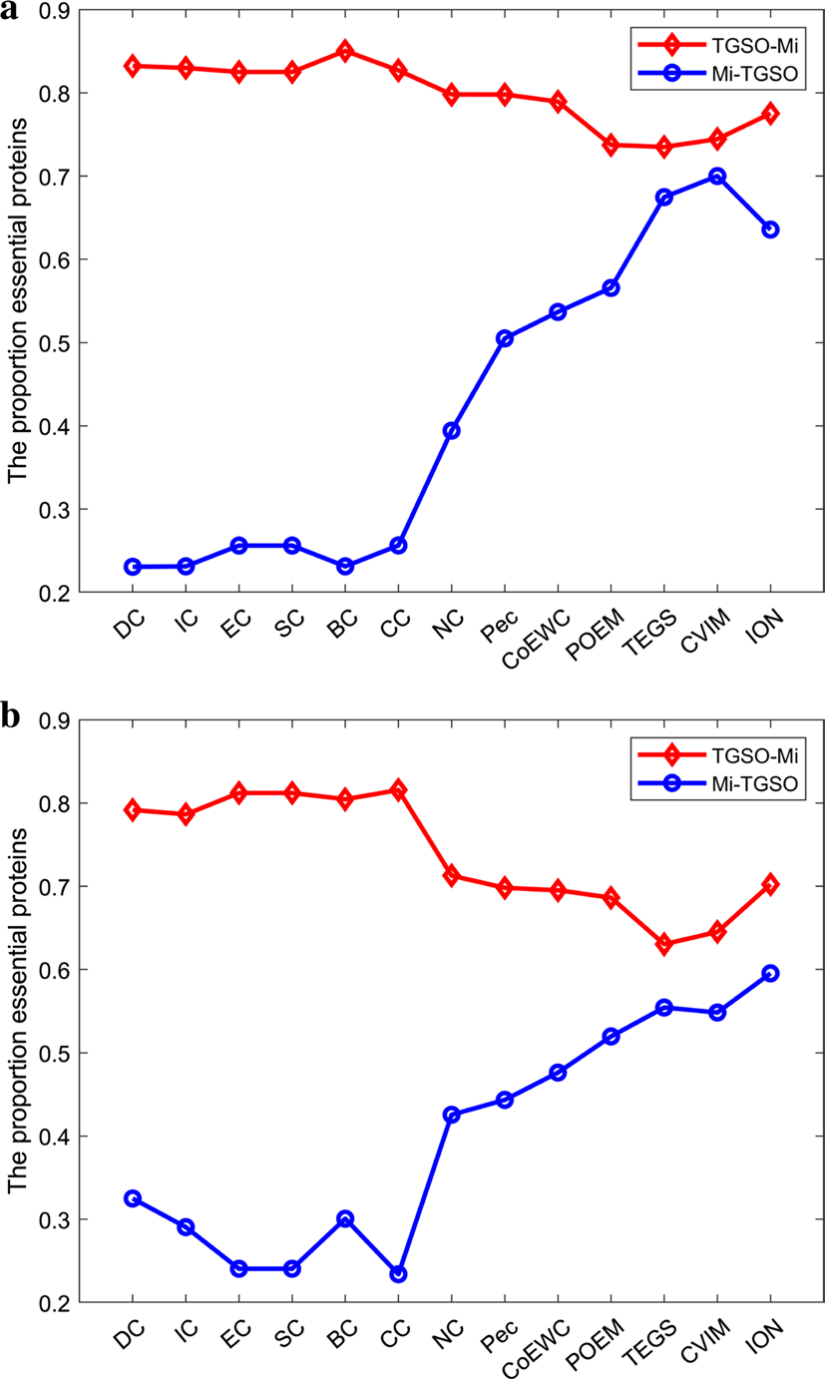
The X-axis represents 13 competing methods. The Y-axis represents the proportion of real key proteins in Mi-TGSO or TGSO-Mi

In order to better reflect the uniqueness and differences between TGSO and these existing competitive methods, we will further compare TGSO with 13 competing prediction models based on the top 200 ranked proteins and the DIP database in this section. And the comparison results are illustrated in Tables 5 and 6. In Tables 5 and 6, $M_i$ represents one of these 13 competitive models, $|TGSO\cap M_i|$ denotes the number of key proteins screened by both TGSO and $M_i$, while $|TGSO-M_i|$ indicates the number of critical proteins found by TGSO instead of $M_i$. From Tables 5 and 6, it can be discovered that TGSO can screen out new key proteins that cannot discovered by any of these 13 competing methods. And in addition, from observing the fourth and fifth columns in both Tables 5 and 6, it can be observed that the proportion of true essential proteins screened by TGSO alone is much higher than the proportion of true essential proteins screened alone by any of these 13 competing methods, which is further demonstrated by the results illustrated in Fig. 9 as well.

**Table 5:** Commonalities and differences between TGSO and 13 competing methods based on the top 200 ranked proteins and the DIP database

Different prediction methods (Mi)	$|TGSO\cap Mi|$	$|TGSO-Mi|$	Percentage of key proteins in ${TGSO-Mi}$ (%)	Percentage of key proteins in ${Mi-TGSO}$ (%)
DC	57	143	83.22	23.08
IC	53	147	82.99	23.13
EC	40	160	82.50	25.63
SC	40	160	82.59	25.61
BC	53	147	85.03	23.13
CC	44	156	82.69	25.64
NC	96	104	79.81	39.42
Pec	101	99	79.80	50.51
CoEWC	105	95	78.95	53.68
POEM	101	99	73.74	56.57
TEGS	117	83	73.49	67.47
CVIM	110	90	74.44	70.00
ION	71	129	77.52	63.57

This table shows the commonalities and differences between TGSO and the 13 competitive methods in Table 1 based on the DIP database

**Table 6:** Commonalities and differences between TGSO and 13 competing methods based on the top 200 ranked proteins and the Krogan database

Different prediction methods (Mi)	$|TGSO\cap Mi|$	$|TGSO-Mi|$	Percentage of key proteins in ${TGSO-Mi}$ (%)	Percentage of key proteins in ${Mi-TGSO}$ (%)
DC	80	120	79.17	32.50
IC	83	117	78.63	29.06
EC	67	133	81.20	24.06
SC	64	136	81.17	24.05
BC	67	133	80.45	30.08
CC	59	141	81.56	23.40
NC	106	94	71.28	42.55
Pec	94	106	69.81	44.34
CoEWC	95	105	69.52	47.62
POEM	98	102	68.63	51.96
TEGS	108	92	63.04	55.43
CVIM	138	62	64.52	54.84
ION	69	131	70.23	59.54

This table shows the commonalities and differences between TGSO and the 13 competitive methods in Table 1 based on the Krogan database

### General applicability of TGSO

In order to prove the applicability of TGSO, we will further execute some simple tests and comparisons based on the Gavin database in this section, and the experimental results are shown in the following Table 7.

**Table 7:** Number of essential proteins predicted by TGSO and 13 methods based on the Gavin database

Methods	Top1% (19)	Top5% (93)	Top10% (196)	Top15% (279)	Top20% (371)	Top25% (464)
SC	0	17	87	130	190	240
EC	0	38	94	134	166	209
BC	9	40	85	122	162	201
DC	7	36	101	158	222	264
IC	16	55	119	163	213	254
CC	11	45	93	135	180	221
NC	11	51	123	170	213	259
PEC	15	69	142	193	238	285
CoEWC	16	69	136	190	237	275
POEM	17	74	148	199	249	296
ION	17	73	150	207	263	312
CVIM	16	80	160	219	271	322
TGSO	19	81	165	221	279	332

This table shows the commonalities and differences between TGSO and the 13 competitive methods in Table 1 based on the Gavin database

As can be seen from Table 7, while comparing with these 13 competing methods, TGSO can achieve the best predictive performance in any range from the top 1% to 25% of potential key proteins, which demonstrates that TGSO is the best prediction model among these competitive models and has wide applicability.

### Effects of parameter on performance of TGSO

In this section, we will analyze the influence of the parameter $\alpha$ on the performance of TGSO. In TGSO, the parameter $\alpha$ with value between 0 and 1 is adopted to adjust the weight of the comprehensive interaction network PCIN and the protein conservatism. During simulation, we will adjust the value of $\alpha$ to study its influence on the performance of TGSO. As shown in Table 8, based on the DIP database, while $\alpha$ is equal to 0.2, the algorithm is in the top 1% and the top 25% respectively takes the maximum value of 48 and 671. When $\alpha$ is 0.4, there are two maximum values of 48 and 487. When $\alpha$ is 0.3, the algorithm reaches the maximum value in the first 1%, the first 10%, and the first 20%. Therefore, on the DIP, 0.3 is the best parameter. In addition, from observing the Table 9, it can be seen that based on the Krogan database, while $\alpha$ varying from 0.1 to 0.4, in the top 1% candidate key proteins, there are $\alpha$ maximum of 35 true essential proteins detected by TGSO, with the accuracy rate of 95%. While $\alpha$ is set to 0.2, TGSO can achieve the best accuracy rate in the top 1% and 25% candidate key proteins. When $\alpha$ is set to 0.3 or 0.4, TGSO achieves the best performance in the two intervals respectively. Therefore, based on the Krogan database, if $\alpha$ is set to 0.2 ,0.3, 0.4, TGSO can achieve the best performance. From Table 10, we can find that when $\alpha$ is between 0.1 and 0.4, only 0.3 occupies two maximum values. To sum up, based on these three kinds of databases, we will set $\alpha$ to 0.3 as the best value in experiments for comparing TGSO with these state-of-the-art competitive models in this article.

**Table 8:** Effects of the parameter $\alpha$ to TGSO based on the DIP database

$\alpha$	0.1	0.2	0.3	0.4	0.5	0.6	0.7	0.8	0.9
Top1%(51)	46	**48**	**48**	**48**	**48**	**48**	47	47	47
Top5%(255)	196	205	208	208	208	208	**209**	202	192
Top10%(510)	336	348	**368**	363	362	354	352	339	330
Top15%(764)	454	483	484	**487**	476	470	466	451	437
Top20%(1019)	558	578	**589**	584	568	556	538	528	528
Top25%(1274)	646	**671**	661	648	644	633	619	610	597

The [bold] indicates the maximum value in the row

This table shows the effects of the parameter $\alpha$ to TGSO based on the DIP database, and the table records the proportion of true key protein in the set of selected proteins

**Table 9:** Effects of the parameter $\alpha$ to TGSO based on the Krogan database

$\alpha$	0.1	0.2	0.3	0.4	0.5	0.6	0.7	0.8	0.9
Top1%(37)	**35**	**35**	**35**	**35**	34	34	34	33	34
Top5%(184)	141	145	147	151	146	146	**153**	145	141
Top10%(367)	242	259	262	262	**264**	262	256	253	242
Top15%(551)	326	350	**364**	362	358	357	349	343	336
Top20%(734)	417	443	447	**449**	438	427	423	413	404
Top25%(918)	502	**524**	515	501	494	493	488	477	469

The [bold] indicates the maximum value in the row

This table shows the effects of the parameter $\alpha$ to TGSO based on the Krogan database, and the table records the proportion of true key protein in the set of selected proteins

**Table 10:** Effects of the parameter $\alpha$ to TGSO based on the Gavin database

$\alpha$	0.1	0.2	0.3	0.4	0.5	0.6	0.7	0.8	0.9
Top1%(19)	17	18	**19**	18	18	18	18	18	18
Top5%(93)	80	82	81	83	83	83	**86**	**86**	79
Top10%(196)	159	163	165	167	167	**169**	167	162	158
Top15%(279)	204	218	221	218	223	**225**	222	216	204
Top20%(371)	247	266	279	**281**	280	280	273	261	255
Top25%(464)	294	304	**332**	326	324	316	311	308	303

The [bold] indicates the maximum value in the row

This table shows the effects of the parameter $\alpha$ to TGSO based on the Gavin database, and the table records the proportion of true key protein in the set of selected proteins

### Albation study

The previous comparative experiments confirmed that TGSO can effectively improve the performance of identifying essential proteins and is superior to existing methods in all aspects. In the design process of TGSO, three kinds of protein interaction networks such as ADN, CEN and CLN were involved from different perspectives. In order to analyze the positive contributions of these networks to the predictive performance of TGSO, we designed the ablation experiment as follows: The initial PPI network is used as the control group, and the experimental groups are ADN, CEN and CLN. All groups are set with the same parameters for iterative calculation, and the optimal result of each group is taken as the representative value of the group. The three evaluation indicators of accuracy, AUC, and F1-score are compared, and the accuracy experimental results obtained are shown in Table 11.

**Table 11:** Model accuracy rates of different networks based on the DIP database

Network	Top1% (51)	Top5% (255)	Top10% (510)	Top15% (764)	Top20% (1019)	Top25% (1274)
*InitPPI*	28	115	239	348	438	533
*ADN*	34	168	294	398	491	570
*CEN*	**46**	**206**	**340**	**452**	**527**	610
*CLN*	41	175	313	444	527	**616**
*PCIN*	**48**	**208**	**368**	**484**	**589**	**661**

The [bold] indicates the maximum value in the row

It can be seen from above Table 11 that in DIP, the initial PPI network contains a lot of noisy data, which leads to poor recognition results. The new network topology of ADN has improved the initial PPI to a certain extent. Among these three kinds of networks, CEN, the protein co-expression network, has a greater improvement in the accuracy of the interval.

**Fig. 10:**
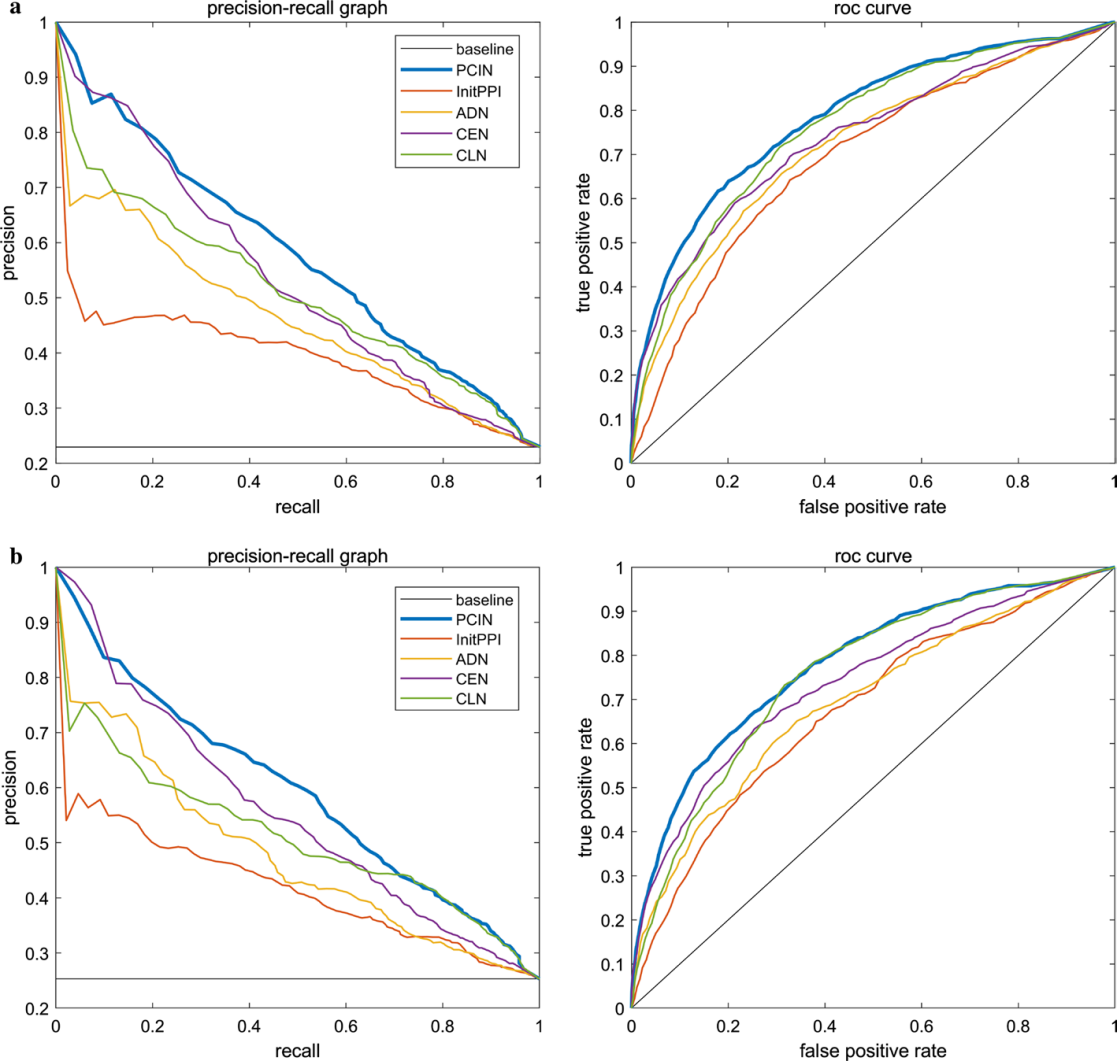
ROC curve and PR curve of various network based on DIP, Krogan,Gavin database. **a** DIP database. **b** Krogan database. **c** Gavin database

In addition, we considered the performance of several networks on the ROC and PR graphs. In the PR chart, the area under the curve of the CEN network was larger than that of other single networks. In the ROC curve chart, CLN performed even better. Through ROC and PR graphs, we calculated the AUC and F1-score values of different network models, detailed results were shown in Table 12 and Fig. 10.

**Table 12:** The AUC and F1-score for all methods in three databases

Method	AUC (DIP)	F1-score (DIP)	AUC (Krogan)	F1-score (Krogan)	AUC (Gavin)	F1-score (Gavin)
*InitPPI*	0.692	0.468	0.678	0.468	0.676	0.588
*ADN*	0.718	0.486	0.695	0.489	0.687	0.597
*CEN*	0.738	0.511	0.739	0.531	0.717	0.610
*CLN*	**0.763**	**0.521**	**0.757**	**0.549**	**0.739**	**0.639**
*PCIN*	**0.785**	**0.555**	**0.781**	**0.560**	**0.760**	**0.647**

The [bold] indicates the maximum value in the row

From observing above Table 12, the obvious based on the DIP database, the AUC value of CLN is 0.763, which is higher than Init (0.692), ADN (0.718), and CEN (0.738). And simultaneously, based on the Krogan and Gavin databases, CLN can achieve the maximum values of AUC and F1-score as well. Therefore, based on above experimental results, we can think that the CLN network, that is, the subcellular colocalization data, may have played the most critical role in the network construction of our prediction model. After analysis, the importance of CLN network is that it can successfully capture characteristics that essential proteins often perform important functions collaboration in the same subcellular location. Therefore, it can provide a positive contribution to the performance of TGSO. In addition, it can be seen as well from the above experimental results that the integrated interaction network PCIN has higher recognition accuracy than any single network, since it can balance the advantages and disadvantages of multiple networks, and eliminate noisy data. Moreover, TGSO can achieve satisfactory performance under multiple evaluation frameworks such as PR graph, ROC graph, AUC and F1-score, which has also fully demonstrated the rationality and excellence of network integration.

## Discussion

Essential proteins are indispensable materials to sustain life activities.In recent years, the development of computational methods for essential protein recognition has become a research hotspot, and many researchers have successively developed various algorithms based on PPI networks. With the gradual improvement of high-throughput biodata, more efficient prediction models have been proposed by combining PPI networks with biodata including the subcellular information and lineal homology information to screen essential proteins. Inspired by this, we first designed a subcellular co-localization score index and a co-expression index based on gene expression data and subcellular data of proteins separately. And then, a novel detection method called TGSO was designed to identify essential proteins based on multiple data fusion. Through comparative experiments, it was confirmed that TGSO is superior to existing methods. Moreover, as for methods including CVIM and TEGS that adopt similar combination of PPI network topology and additional biological information with TGSO, although the numbers of essential proteins in top 200 ranked proteins are similar, but the detailed essential proteins detected by TGSO is very different from that detected by TEGS and CVIM. During experiment, we tried to combine features selected by these models with features in TGSO, but experimental results showed that the recognition effect of fusing these features is not ideal. Through analysis, this might be caused by that the criticality of key proteins is very diverse. For example, in TEGS, the importance of protein was predicted by combining GO annotation with homologous prediction and subcellular localization data. But many GO annotations were provided on the basis of orthology predictions, i.e. an annotation was provided in one species based on published experimental evidence. Hence, the same annotation was transferred to the orthologous proteins. If the term did not exclude homologous transferred by predicted orthology, it would make TEGS become highly redundant. In CVIM, gene expression and network topology information were adopted, but the subcellular location information was not considered. And moreover, the entropy weighted method was only used to integrate topological features, however, topological features often have lots of noisy data, so the effect of CVIM would be limited. In general, TGSO can achieve better predictive performance. In the future, we will carry out a more in-depth analysis of it, and look for better characteristic information to collect key proteins found by different methods and improve the recognition rate of TGSO.

## Conclusions

In this paper, we propose a new prediction model:TGSO. In TGSO, DBN is introduced to construct the node aggregation degree interactive network (ADN), PCC is adopted to construct the protein co-expression interactive network (CEN), and the subcellular localization information is adopted to construct the protein co-localization interactive network (CLN) firstly. And then, by integrating these three kinds of interactive networks, a comprehensive protein interaction network (PCIN) is obtained. Next, through combining protein conservatism scores with the PCIN, an iterative algorithm is proposed to calculate the essentiality score for each protein, which can be used to screen essential proteins efficiently. Finally, intensive experiments have been conducted to estimate the performance of TGSO based on the DIP, Krogan and Gavin databases separately, and experimental results show that TGSO can achieve more satisfactory performance than traditional state-of-the-art methods. In future work, we will introduce more biological information such as the protein–domain interactions and the gene ontology information to further improve the prediction performance of TGSO.

## Data Availability

The datasets used and/or analyzed during the current study are available from the first author or corresponding author on reasonable request.

## Abbreviations

BC: Betweenness Centrality
CC: Closeness Centrality
CoEWC: Co-Expression Weighted by Clustering coefficient
DC: Degree Centrality
EC: Eigenvector Centrality
IC: Information Centrality
NC: Neighbor Centrality
PPI: Protein–Protein Interaction
SC: Subgraph Centrality
RWHN: Randomly Walking in the Heterogeneous Network
DBN: Density between nodes
ADC: Aggregation degree interaction network
CEN: Co-expression interaction network; Co-localization interaction netwrok
PCIN: Protein comprehensive interaction network

## References

[1] Roemer T, Jiang B, Davison J, Ketela T, Veillette K, Breton A, Tandia F, Linteau A, Sillaots S, Marta C (2003). Large-scale essential gene identification in *Candida albicans* and applications to antifungal drug discovery. Mol Microbiol.

[2] Zhang Z, Wu FX, Wang J, Qi L, Zheng R, Min L (2016). Prioritizing disease genes by using search engine algorithm. Curr Bioinform.

[3] Glass JI, Iii CH, Smith HO, Venter JC (2014). A systems biology tour de force for a near-minimal bacterium. Mol Syst Biol.

[4] Steinmetz LM, Scharfe C, Deutschbauer AM, Mokranjac D, Herman ZS, Jones T, Chu AM, Giaever G, Prokisch H, Oefner PJ (2002). Systematic screen for human disease genes in yeast. Nat Genet.

[5] Cullen LM, Arndt GM (2005). Genome-wide screening for gene function using rnai in mammalian cells. Immunol Cell Biol.

[6] Kamath RS, Fraser AG, Dong Y, Poulin G, Durbin R, Gotta M, Kanapin A, Le Bot N, Moreno S, Sohrmann M (2003). Systematic functional analysis of the *Caenorhabditis elegans* genome using rnai. Nature.

[7] Giaever G, Chu AM, Li N, Connelly C, Johnston M (2002). Functional profiling of the saccharomyces cerevisiae genome. Nature.

[8] Lei C, Ge X, Ping X (2015). Identifying essential *Streptococcus sanguinis* genes using genome-wide deletion mutation. Methods Mol Biol.

[9] Ji Y, Zhang B, Van SF, Warren P, Woodnutt G, Burnham MK, Rosenberg M (2001). Identification of critical staphylococcal genes using conditional phenotypes generated by antisense rna. Science.

[10] Gallagher LA, Ramage E, Jacobs MA, Kaul R, Brittnacher M, Manoil C (2007). A comprehensive transposon mutant library of *Francisella novicida*, a bioweapon surrogate. Proc Natl Acad Sci.

[11] Langridge GC, Phan M-D, Turner DJ, Perkins TT, Parts L, Haase J, Charles I, Maskell DJ, Peters SE, Dougan G (2009). Simultaneous assay of every *Salmonella typhi* gene using one million transposon mutants. Genome Res.

[12] Yu H, Kim PM, Sprecher E, Trifonov V, Gerstein M (2007). The importance of bottlenecks in protein networks: correlation with gene essentiality and expression dynamics. PLoS Comput Biol.

[13] Li M, Wang J, Chen X, Wang H, Pan Y (2011). A local average connectivity-based method for identifying essential proteins from the network level. Comput Biol Chem.

[14] Li M, Lu Y, Wang J, Wu F-X, Pan Y (2014). A topology potential-based method for identifying essential proteins from PPI networks. IEEE/ACM Trans Comput Biol Bioinform.

[15] Jeong HM, Mason SP, Barabási A, Oltvai ZN (2001). Lethality and centrality in protein networks. Nature.

[16] Hahn MW, Kern AD (2005). Comparative genomics of centrality and essentiality in three eukaryotic protein-interaction networks. Mol Biol Evol.

[17] Zelen SM (1989). Rethinking centrality: methods and examples. Soc Netw.

[18] Bonacich P (1987). Power and centrality: a family of measures. Am J Sociol.

[19] Estrada E, Rodriguez-Velazquez JA (2005). Subgraph centrality in complex networks. Phys Rev E Stat Nonlinear Soft Matter Phys.

[20] Joy MP, Brock A, Ingber DE, Sui H (2014). High-betweenness proteins in the yeast protein interaction network. J Biomed Biotechnol.

[21] Wuchty S, Stadler PF (2003). Centers of complex networks. J Theor Biol.

[22] Wang J, Li M, Wang H, Pan Y (2011). Identification of essential proteins based on edge clustering coefficient. IEEE/ACM Trans Comput Biol Bioinform.

[23] Kuchaiev O, Rašajski M, Higham DJ, Pržulj N, Przytycka TM (2009). Geometric de-noising of protein–protein interaction networks. PLoS Comput Biol.

[24] Sprinzak E, Sattath S, Margalit H (2003). How reliable are experimental protein–protein interaction data?. J Mol Biol.

[25] Min L, Zhang H, Wang JX, Yi P (2012). A new essential protein discovery method based on the integration of protein–protein interaction and gene expression data. BMC Syst Biol.

[26] Xue Z, Xu J, Xiao WX (2013). A new method for the discovery of essential proteins. PLoS ONE.

[27] Zhao B, Wang J, Li M, Wu FX, Pan Y (2014). Prediction of essential proteins based on overlapping essential modules. IEEE Trans NanoBiosci.

[28] Zhao B, Zhao Y, Zhang X, Zhang Z, Wang L (2019). An iteration method for identifying yeast essential proteins from heterogeneous network. BMC Bioinform.

[29] Ashburner M, Ball CA, Blake JA, Botstein D, Butler H, Cherry JM, Davis AP, Dolinski K, Dwight SS, Eppig JTA (2000). Gene ontology: tool for the unification of biology. Nat Genet.

[30] Kim Wooyoung (2012). Prediction of essential proteins using topological properties in go-pruned PPI network based on machine learning methods. Tsinghua Sci Technol.

[31] Zhang Z, Luo Y, Hu S, Li X, Wang L, Zhao B (2020). A novel method to predict essential proteins based on tensor and hits algorithm. Hum genom.

[32] Lei X, Yang X, Wu F-X (2018). Artificial fish swarm optimization based method to identify essential proteins. IEEE/ACM Trans Comput Biol Bioinform.

[33] Zhang W, Xu J, Li Y, Zou X (2016). Detecting essential proteins based on network topology, gene expression data, and gene ontology information. IEEE/ACM Trans Comput Biol Bioinform.

[34] Zhang W, Xu J, Zou X (2019). Predicting essential proteins by integrating network topology, subcellular localization information, gene expression profile and go annotation data. IEEE/ACM Trans Comput Biol Bioinform.

[35] Lei X, Zhao J, Fujita H, Zhang A (2018). Predicting essential proteins based on rna-seq, subcellular localization and go annotation datasets. Knowl Based Syst.

[36] Zhang X, Xiao W, Xiao W (2020). Deephe: accurately predicting human essential genes based on deep learning. PLOS Comput Biol.

[37] Wang N, Zeng M, Zhang J, Li Y, Li M. Ess-NEXG: predict essential proteins by constructing a weighted protein interaction network based on node embedding and XGBoost. Bioinform Res Appl (2020)

[38] Peng W, Wang J, Wang W, Liu Q, Wu F-X, Pan Y (2012). Iteration method for predicting essential proteins based on orthology and protein–protein interaction networks. BMC Syst Biol.

[39] Zhang X, Xiao W, Hu X (2018). Predicting essential proteins by integrating orthology, gene expressions, and ppi networks. PLoS ONE.

[40] Lei X, Yang X (2018). A new method for predicting essential proteins based on participation degree in protein complex and subgraph density. PLoS ONE.

[41] Li G, Li M, Wang J, Li Y, Pan Y. United neighborhood closeness centrality and orthology for predicting essential proteins. IEEE/ACM Trans Comput Biol Bioinform 1–1 (2018) 10.1109/TCBB.2018.2889978 30596582

[42] Zhang F, Peng W, Yang Y, Dai W, Song J (2019). A novel method for identifying essential genes by fusing dynamic protein–protein interactive networks. Genes.

[43] Li S, Chen Z, He X, Zhang Z, Wang L (2020). An iteration method for identifying yeast essential proteins from weighted ppi network based on topological and functional features of proteins. IEEE Access.

[44] Pereira-Leal JB, Audit B, Peregrin-Alvarez JM, Ouzounis CA (2005). An exponential core in the heart of the yeast protein interaction network. Mol Biol Evol.

[45] Horyu D, Hayashi T (2013). Comparison between Pearson correlation coefficient and mutual information as a similarity measure of gene expression profiles. Jpn J Biom.

[46] Hart GT, Lee I, Marcotte EM (2007). A high-accuracy consensus map of yeast protein complexes reveals modular nature of gene essentiality. BMC Bioinform.

[47] Dezső Z, Oltvai ZN, Barabási A-L (2003). Bioinformatics analysis of experimentally determined protein complexes in the yeast saccharomyces cerevisiae. Genome Res.

[48] Zotenko E, Mestre J, O’Leary DP, Przytycka TM. Why do hubs in the yeast protein interaction network tend to be essential: reexamining the connection between the network topology and essentiality. PLoS Comput Biol. 2008;4(8):1000140. 10.1371/journal.pcbi.1000140 PMC2467474 18670624

[49] Kumar A, Agarwal S, Heyman JA, Matson S, Heidtman M, Piccirillo S, Umansky L, Drawid A, Jansen R, Liu Y (2002). Subcellular localization of the yeast proteome. Genes Dev.

[50] Schwikowski B, Uetz P, Fields S (2000). A network of protein–protein interactions in yeast. Nat Biotechnol.

[51] Page L, Brin S, Motwani R, Winograd T. The pagerank citation ranking: bringing order to the web. Technical report, Stanford InfoLab (1999).

[52] Xenarios I, Salwinski L, Duan XJ, Higney P, Kim S-M, Eisenberg D (2002). Dip, the database of interacting proteins: a research tool for studying cellular networks of protein interactions. Nucleic Acids Res.

[53] Zhong G, Guo X, Ignatchenko A, Li J, Pu S, Datta N, Tikuisis AP, Krogan NJ, Cagney G, Yu H (2006). Global landscape of protein complexes in the yeast saccharomyces cerevisiae. Nature.

[54] Gavin A-C, Aloy P, Grandi P, Krause R, Boesche M, Marzioch M, Rau C, Jensen LJ, Bastuck S, Dümpelfeld B (2006). Proteome survey reveals modularity of the yeast cell machinery. Nature.

[55] Mewes H-W, Frishman D, Mayer KF, Münsterkötter M, Noubibou O, Pagel P, Rattei T, Oesterheld M, Ruepp A, Stümpflen V (2006). Mips: analysis and annotation of proteins from whole genomes in 2005. Nucleic Acids Res.

[56] Cherry JM, Adler C, Ball C, Chervitz SA, Dwight SS, Hester ET, Jia Y, Juvik G, Roe T, Schroeder M (1998). Sgd: saccharomyces genome database. Nucleic Acids Res.

[57] Saccharomyces Genome Deletion Project. http://yeastdeletion.stanford.edu/.

[58] Zhang R, Lin Y (2009). Deg 50, a database of essential genes in both prokaryotes and eukaryotes. Nucleic Acids Res.

[59] Tu BP, Kudlicki A, Rowicka M, McKnight SL (2005). Logic of the yeast metabolic cycle: temporal compartmentalization of cellular processes. Science.

[60] Östlund G, Schmitt T, Forslund K, Köstler T, Messina DN, Roopra S, Frings O, Sonnhammer EL (2010). Inparanoid 7: new algorithms and tools for eukaryotic orthology analysis. Nucleic Acids Res.

[61] Peng X, Wang J, Zhong J, Luo J, Pan Y. An efficient method to identify essential proteins for different species by integrating protein subcellular localization information. In: 2015 IEEE international conference on bioinformatics and biomedicine (BIBM), pp 277–280 (2015). IEEE.

[62] Binder JX, Pletscher-Frankild S, Tsafou K, Stolte C, O’Donoghue SI, Schneider R, Jensen LJ. Compartments: unification and visualization of protein subcellular localization evidence. Database 2014 (2014). 10.1093/database/bau012 PMC3935310 24573882

[63] Holman AG, Davis PJ, Foster JM, Carlow CK, Kumar S (2009). Computational prediction of essential genes in an unculturable endosymbiotic bacterium, wolbachia of brugia malayi. BMC Microbiol.

[64] Ping P, Wang L, Kuang L, Ye S, Iqbal MFB, Pei T (2018). A novel method for lncRNA-disease association prediction based on an lncRNA-disease association network. IEEE/ACM Trans Comput Biol Bioinform.

[65] Li J, Li X, Feng X, Wang B, Zhao B, Wang L (2019). A novel target convergence set based random walk with restart for prediction of potential lncRNA-disease associations. BMC Bioinform.

[66] Chen Z, Meng Z, Liu C, Wang X, Kuang L, Pei T, Wang L (2020). A novel model for predicting essential proteins based on heterogeneous protein-domain network. IEEE Access.

[67] Davis J. The relationship between precision–recall and roc curves. In: Proceedings of the 23th international conference on machine learning, 2006 (2006).

